# Risk stratification of prostate cancer with MRI and prostate-specific antigen density-based tool for personalized decision making

**DOI:** 10.1093/bjr/tqad027

**Published:** 2023-12-12

**Authors:** Ishwariya Rajendran, Kang-Lung Lee, Liness Thavaraja, Tristan Barrett

**Affiliations:** Department of Radiology, Addenbrooke’s Hospital and University of Cambridge, Cambridge CB2 0QQ, United Kingdom; Department of Radiology, Addenbrooke’s Hospital and University of Cambridge, Cambridge CB2 0QQ, United Kingdom; Department of Radiology, Taipei Veterans General Hospital, Taipei 11217, Taiwan; School of Medicine, National Yang Ming Chiao Tung University, Taipei 112304, Taiwan; School of Medicine, Addenbrooke’s Hospital, Cambridge CB2 0SP, United Kingdom; Department of Radiology, Addenbrooke’s Hospital and University of Cambridge, Cambridge CB2 0QQ, United Kingdom

**Keywords:** prostate cancer, MRI, risk assessment

## Abstract

**Objectives:**

MRI is now established for initial prostate cancer diagnosis; however, there is no standardized pathway to avoid unnecessary biopsy in low-risk patients. Our study aimed to test previously proposed MRI-focussed and risk-adapted biopsy decision models on a real-world dataset.

**Methods:**

Single-centre retrospective study performed on 2055 biopsy naïve patients undergoing MRI. Diagnostic pathways included “biopsy all”, “MRI-focussed” and two risk-based MRI-directed pathways. Risk thresholds were based on prostate-specific antigen (PSA) density as low (<0.10 ng mL^−2^), intermediate (0.10-0.15 ng mL^−2^), high (0.15-0.20 ng mL^−2^), or very high-risk (>0.20 ng mL^−2^). The outcome measures included rates of biopsy avoidance, detection of clinically significant prostate cancer (csPCa), missed csPCa, and overdiagnosis of insignificant prostate cancer (iPCa).

**Results:**

Overall cancer rate was 39.9% (819/2055), with csPCa (Grade-Group ≥2) detection of 30.3% (623/2055). In men with a negative MRI (Prostate Imaging-Reporting and Data System, PI-RADS 1-2), the risk of cancer was 1.2%, 2.6%, 9.0%, and 12.9% in the low, intermediate, high, and very high groups, respectively; for PI-RADS score 3 lesions, the rates were 10.5%, 14.3%, 25.0%, and 33.3%, respectively. MRI-guided pathway and risk-based pathway with a low threshold missed only 1.6% csPCa with a biopsy-avoidance rate of 54.4%, and the risk-based pathway with a higher threshold avoided 62.9% (1292/2055) of biopsies with 2.9% (61/2055) missed csPCa detection. Decision curve analysis found that the “risk-based low threshold” pathway has the highest net benefit for probability thresholds between 3.6% and 13.9%.

**Conclusion:**

Combined MRI and PSA-density risk-based pathways can be a helpful decision-making tool enabling high csPCa detection rates with the benefit of biopsy avoidance and reduced iPCa detection.

**Advances in knowledge:**

This real-world dataset from a large UK-based cohort confirms that combining MRI scoring with PSA density for risk stratification enables safe biopsy avoidance and limits the over-diagnosis of insignificant cancers.

## Introduction

Multiparametric MRI is now established as the initial diagnostic test for men presenting with suspected non-metastatic prostate cancer.^1^ The Prostate Imaging-Reporting and Data System (PI-RADS) guidelines have been an important step in standardizing the technical specifications for MR acquisition and the approach to reporting studies.^2^ The high negative predictive value of MRI enables biopsy avoidance in men considered of low risk.^3^ Nevertheless, there remains variability in practice for both approach to biopsy and selection for biopsy, particularly for men with low probability of equivocal MRI findings.^4–7^ The decision to biopsy will be further impacted by patient age and co-morbidity, and whether patients and/or clinicians are considered to be “biopsy adverse” or “cancer adverse” and prioritizing all cancer detection of any grade.^8^

A recent Cochrane systematic review and meta-analysis supports the MRI-directed biopsy pathway wherein patients with PI-RADS score 1-2 MRIs do not undergo biopsy and PI-RADS scores 3-5 undergo an MRI-directed biopsy, which will avoid biopsy in 33% of men and reduces the detection of insignificant prostate cancer (iPCa) by 10%.^9^ However, 45% of men with PI-RADS MRI score ≥3 do not have significant cancer,^10^ suggesting the need for better patient stratification with risk models based on the incorporation of additional clinical information.

Several risk prediction models have been proposed, with the aim of avoiding unnecessary prostate biopsies in patients that are either likely to have benign disease or to harbour clinically iPCa. Recently, the simplistic combination of PSA-density thresholds with MRI-derived PI-RADS scoring has been shown to outperform these more complex models.^11^ This led Schoots et al. to develop a risk-adapted pathway for biopsy decision making based on MRI findings and the current urological guidelines for PSA-density thresholds,^10^ initially developed as a hypothesis-generating approach and in need of external validation. At our institution, we have performed prostate MRI in biopsy naïve patients since 2015, with a prospectively maintained database of >2000 patients. The aim of our study was to test the proposed MRI focussed and risk-adapted biopsy decision models on this real-world dataset.

## Methods

This single-centre retrospective analysis was performed on patients undergoing prostate MRI from November 2015 to October 2021, with the need for informed consent for data analysis waived by the Local Ethics Committee (IRAS 313163). Consecutive patients referred for multiparametric MRI with a suspicion of having localized or locally advanced prostate cancer based on raised prostate-specific antigen (PSA) levels and/or positive digital rectal examination were included. Exclusion criteria included the inability to accurately assign an MRI score due to significant associated artefact (*n* = 37), biopsy performed prior to MRI (*n* = 5), prior history of prostate cancer (*n* = 10), and previous treatment for benign prostatic conditions (*n* = 22). A total of 2055 patients were evaluated, with outcome measures including rates of biopsy avoidance, detection rate of csPCa, missed csPCa, and overdiagnosis of iPCa.

### Diagnostic pathways

The outcomes of the following diagnostic pathways were analysed: biopsy for all patients, an MRI-focussed pathway and two risk-based (MRI-directed) pathways using low or high thresholds (Table 1). The PSA density (PSAD) was categorized into being low risk when <0.10 ng mL^−2^, intermediate 0.10-0.15 ng mL^−2^, high 0.15-0.20 ng mL^−2^, or very high risk when >0.20 ng mL^−2^.

**Table 1.** Diagnostic pathway model.

Diagnostic pathway	Biopsy criteria	Selection for biopsy	
		MRI score	PSAD
Biopsy all	All men with suspected PCa irrespective of MRI findings or PSAD	Not considered	Not considered
MRI-focused	MRI based	≥PI-RADS 3	Not considered
Risk-based low threshold pathway	MRI and PSAD based	PI-RADS 1-2	>0.20 ng mL^−2^
		PI-RADS 3	≥0.10-0.15 ng mL^−1^
		PI-RADS 4-5	All
Risk-based high threshold pathway	MRI and PSAD based	PI-RADS 3	>0.20 ng mL^−2^
		PI-RADS 4-5	All

### MRI

Patients underwent prostate MRI on either a 1.5 T (15%) MR450 or 3.0 T (85%) Discovery MR750 HDx (GE Healthcare, Waukesha/Chicago, IL, USA) with a 16-32 channel surface phased array coil. The protocol has been described in detail previously^12^; in brief, this comprised axial Fast Spin Echo T1-weighted images of the pelvis, along with T2-weighted Fast Recovery Fast Spin Echo images of the prostate acquired in the axial (slice thickness 3 mm; gap 0-1 mm), sagittal and coronal planes (Table 2). Axial diffusion-weighted imaging (DWI) was performed using a dual spin-echoplanar imaging pulse sequence with slice thickness 3-4 mm; gap 0 mm, and automated apparent diffusion coefficient maps. An additional small-FOV (24 × 12 cm) DWI series was performed at 1400 s mm^−2^ (1.5 T) or 2000 s mm^−2^ (3 T). Dynamic contrast-enhanced (DCE)-MRI was acquired as an axial 3D fast spoiled gradient echo sequence following bolus injection of Gadobutrol (Gadovist, Bayer HealthCare) via a power injector at a rate of 3 mL s^−1^ (dose 0.1 mmol kg^−1^) followed by a 25 mL saline flush, injection at 28 s/40 s, temporal resolution 7 s/10 s. The axial T2, DWI, and DCE sequences were matched in orientation, slice thickness, and gap.

**Table 2.** MRI protocol.

Parameter	T1 TSE	T2 TSE	epi-2D	DCE
TR ms/TE ms	561/11	4273/102	3775/70	4.1/1.8
Flip angle (°)	70	111	90	13
ETL length/Epi-factor	4	16	1	x
Averages	2	1.5	6	x
b-Value	x	x	150, 750, 1400	x
Section thickness (mm)	8	3	3-4	3
FOV (mm)	240	220	280	240
Resolution	1.1 × 1.0	0.7 × 0.5	2.2 × 2.2	1.2 × 1.2
Acquisition time (min)	03:43	06:59	02:58	05:57

### Image analysis

Images were reviewed by specialist uroradiologists with 6-13 years’ experience and considered experts based on number of MRIs reported.^13^^,^^14^ MRI sequences were evaluated based on the PI-RADS structured scoring criteria, with weighting applied for T2WI and DWI scoring depending on the peripheral zone (PZ) or transition zone (TZ) location of lesions following the dominant sequence paradigm.^15^ All clinical information was available at the time of reporting, performed as part of standard clinical care.

### Biopsy

Transrectal or transperineal approach biopsies were performed by experienced urologists based on clinical recommendations. Two to four target cores were obtained from lesions with PI-RADS score 3-5 using MRI-ultrasound fusion, with additional systematic cores obtained. UroNav (InVivo Corp., Gainesville, FL, USA) was used for transrectal biopsy (with 12 systematic cores) and Biopsee (Medcom, Darmstadt, Germany) was used for transperineal biopsy (with 24 systematic cores).^16^^,^^17^ Pre-biopsy delineation of lesions (PI-RADS 3-5) was performed by radiologists using DynaCAD (InVivo Corp.). For cases with a negative MRI, the decision for biopsy was taken on a case-by-case basis following discussion with the patient and clinical risk assessment, predominantly based on family history, absolute PSA threshold of >10 ng mL^−2^, and/or a PSA density threshold of >0.15 ng mL^−2^. Patients with negative MRI and no biopsy were followed for a minimum of 6 months and had at least one subsequent PSA reading.

### Histopathology

All biopsies were graded with a Gleason score according to the International Society of Urological Pathology (ISUP) 2005 recommendations by a specialist uropathologist. Cases were then reviewed by another uropathologist in a multidisciplinary team meeting. The final result was used for outcome analysis of the study. Patients with Gleason score 3 + 3 (ISUP group 1) were classified as iPCa and Gleason score ≥3 + 4 (ISUP group ≥2) as csPCa.

### Statistics

Medians with IQR were calculated for continuous variables. Outcomes were measured in terms of the rate of csPCa detection, iPCa detection, potential avoided biopsies, missed csPCa, and negative biopsies. Pearson’s chi-square test was used to assess the differences in categorical variables. A decision curve analysis (DCA) was used to evaluate the clinical “net benefit” of the diagnostic pathway.^18^^,^^19^ The DCA was calculated by subtracting the harm of conducting unnecessary biopsy (false positive) concerning threshold probability (p_t_) from the benefit of detecting significant cancer (true positive),^19^ where *N* is the number of total subjects in a diagnostic pathway, formulated as:

$$\mathrm{Net}\;\mathrm{benefit}=\;\frac{\mathrm{True}\;\mathrm{Positives}}{N}-\frac{\mathrm{False}\;\mathrm{Positives}}{N}\;x\;\frac{p_{t}}{1-p_{t}}$$

R 4.2.0 (R Foundation for Statistical Computing, Vienna, Austria) was used to conduct statistical analysis. DCA was performed using rmda 1.65, a package of R.

## Results

A total of 2055 patients were evaluated, with a median age of 65 years (IQR: 59-69 years) and median PSA of 6.31 (IQR 4.65-9.12) ng mL^−2^ (Table 3). PI-RADS 1-2 MRI scores were reported in 54.2% of patients (1113/2055), PI-RADS 3 in 10.0% (206/2055), and PI-RADS 4-5 in 35.8% (736/2055). A total of 1158 patients underwent biopsy. The total number of cancers in the cohort was 39.9% (819/2055), with the prevalence of csPCa (ISUP ≥2) being 30.3% (623/2055). The 897 men with PI-RADS score 1-3 who did not undergo biopsy had a median follow-up of 33 months (IQR 14—51 months) and were considered to be negative for csPCa.

**Table 3.** Characteristics of the patient cohort.

Age (years)	65 (59-69)
PSA (ng mL^−1^)	6.31 (4.65-9.12)
Prostate volume (cm^3^)	54.7 (38-79)
PSA density (ng mL^−2^)	0.11 (0.08-0.17)
• PSA density <0.10 ng mL^−2^	790 (38.4%)
• PSA density 0.10-0.15 ng mL^−2^	576 (28.0%)
• PSA density 0.15-0.20 ng mL^−2^	268 (13.1%)
• PSA density >0.20 ng mL^−2^	421 (20.4%)
Gleason score	
• Group 1 (GS 3 + 3)	196 (9.5%)
• Group 2 (GS 3 + 4)	339 (16.5%)
• Group 3 (GS 4 + 3)	124 (6.0%)
• Group 4 (GS 8)	47 (2.3%)
• Group 5 (GS 9-10)	113 (5.5%)
MRI score	
• PI-RADS 1	15 (0.7%)
• PI-RADS 2	1098 (53.5%)
• PI-RADS 3	206 (10.0%)
• PI-RADS 4	238 (11.6%)
• PI-RADS 5	498 (24.2%)

Values reported as median with IQR in parentheses.

Table 4 highlights the prevalence of cancer relative to each category of PI-RADS and by PSAD groups. In men with a negative MRI (PI-RADS 1 and 2), the risk of cancer was 1.2% in the low-risk PSA density group, 2.6% in intermediate, and 9.0% in the high group. However, for the very-high PSA density group the risk was 12.9%. In men with indeterminate PI-RADS 3 lesions, the risk was 10.5%, 14.3%, 25.0%, and 33.3% in the low, intermediate, high, and very high-risk PSA density groups, respectively. The risk ranged from 44.6% to 89.5% in all men with PIRADS 4 or 5 lesions (Table 4).

**Table 4.** Analysis of risk-based pathway in our population.

	csPCa prevalence relative to PI-RADS score	csPCa prevalence in the PSA-density risk groups			
**Total**	**ISUP ≥2 Ca prevalence**	**Low (<0.10)**	**Intermediate-low (0.10-0.15)**	**Intermediate-high (0.15-0.20)**	**High (≥0.20)**
**ALL PI-RADS**	623/2055 (30.3%)	65/790 (8.2%)	133/576 (23.1%)	118/269 (43.9%)	307/420 (73.1%)
**PI-RADS 1-2**	34/1113 (3.1%)	7/602 (1.2%)	9/341 (2.6%)	9/100 (9.0%)	9/70 (12.9%)
**PI-RADS 3**	36/206 (17.5%)	8/76 (10.5%)	9/63 (14.3%)	10/40 (25.0%)	9/27 (33.3%)
**PI-RADS 4-5**	553/736 (75.1%)	**50/112 (44.6%)**	**115/172 (66.9%)**	**99/129 (76.7%)**	**289/323 (89.5%)**

Very low	0%-5% csPCa (below population risk)	No biopsy
Low	5%-10% csPCa (acceptable risk)	No biopsy
Intermediate-low	10%-20% csPCa	Consider biopsy
Intermediate-high	20%-30% csPCa	Highly consider biopsy
High	30%-40% csPCa	Perform biopsy
**Very high**	**>40% csPca**	**Perform biopsy**

## Outcome analysis

### “Biopsy all” pathway

This formed the comparator group, wherein no csPCa would be missed. The clinically significant cancer detection rate was 30.3% (623/2055), with a 9.5% detection of clinically insignificant cancer and 60.1% of biopsies being negative (Table 5).

**Table 5:** Pathway outcome.

Pathway	Patients (*n*)	Cancer detection rate				Missed csPCa	*P*-value	Biopsy avoidance	*P*-value	Avoided iPCa	Negative biopsy	*P*-value
		csPCa	*P*-value	iPCa	*P*-value							
Biopsy for all	2055 (100%)	30.3% (623)	–	9.5% (196)	–	None	–	N/A	–	N/A	60.1% (1236)	–
MRI guided pathway	2055 (100%)	28.7% (589)	.245	7.3% (150)	.010	1.7% (34)	<.001	54.2% (1113)	<.001	2.2% (46)	17.2% (353)	<.001
Risk based pathway with low threshold	2055 (100%)	28.7% (590)	.259	7.3% (151)	.012	1.6% (33)	<.001	54.5% (1119)	<.001	2.2% (45)	16.9% (346)	<.001
Risk based pathway with high threshold	2055 (100%)	27.3% (562)	.036	5.8% (120)	<.001	2.9% (61)	<.001	62.9% (1292)	<.001	3.7% (76)	9.8% (201)	<.001

### MRI-guided pathway

Following this pathway, the csPCa cancer detection rate was 28.7% (589/2055), with an iPCa detection rate of 7.3% (150/2055). The biopsy avoidance rate was 54.2% (1113/2055), with a reduction in iPCa detection of 2.2% (46/2055) at the expense of csPCa being missed in 1.7% (34/2055).

### Risk-based low-threshold pathway

Clinically significant cancer was detected in 28.7% (590/2055) and iPCa in 7.3% (151/2055), with 64.9% having a benign outcome (1314/2055). Biopsy would be avoided in 54.5% (1119/2055), with 1.6% of csPCa missed (33/2055) and a 2.2% (45/2055) reduction in iPCa detection.

### Risk-based high-threshold pathway

The pathway would have detected 27.3% (562/2055) of csPCa, with a 5.8% diagnosis of iPCa (120/2055) and benign outcome for 66.8% (1373/2055) men. Clinically significant PCa would have been missed in 2.9% (61/2055), with a 3.7% (76/2055) detection of iPCa and biopsy avoidance was increased by 19.3% to a rate of 62.9% (1292/2055).

**Figure 1.**
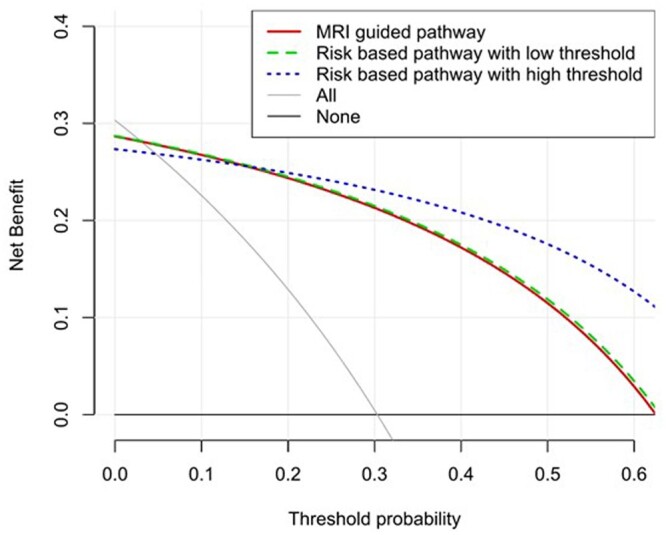
DCA for net benefit for a range of probability thresholds from 0 to 0.6. The x-axis of the decision curve denotes threshold probability, defined as the minimum probability of disease at which a decision-maker would opt for a biopsy. The lower extreme on the *x*-axis (ie, threshold probability) reflects a “cancer averse” situation, whilst the higher extreme represents a “biopsy averse” scenario. The *y*-axis represents the net benefit of a pathway.

In the decision curve analysis, the “biopsy all” strategy had the highest net benefit for probability thresholds between 0% and 3.6%. The “risk-based low threshold pathway” shows the highest net benefit for probability thresholds between 3.6% and 13.9%. When probability thresholds are greater than 13.9%, the “risk-based high threshold pathway” dominated with the highest net benefit (Figure 1).

## Analysis based on PI-RADS v2.1 publication date

A sub-analysis based on the publication date of PI-RADS v2.1 was performed. The prevalence of csPCa was 29.1% (317/1089) during the PI-RADS v2.0 era (up to 31/03/2019), and 31.7% (306/966) in the PI-RADS v2.1 era (after 01/04/2019). When categorizing patients according to MRI PI-RADS scores and PSAD risk groups, a similar pattern was noted regarding biopsy recommendations across both cohorts (Supplementary Tables 1 and 2). Noteworthy, minor differences were observed in the PI-RADS v2.1 era, wherein negative MRIs with high PSAD values decreased from the intermediate-low risk group (19.4%) to the low-risk category (5.9%) and indeterminate PI-RADS 3 cases with low PSAD values demonstrated a downgrade from the intermediate-low risk group (11.9%) to the low-risk category (8.8%) (Supplementary Table 2).

## Discussion

In this study, we compared the outcomes of four proposed diagnostic pathways for investigating men with suspected PCa. We found that the risk-based “low” threshold pathway and MRI-focused pathways have the highest cancer detection rate. They also show a potential reduction in overdiagnosis of iPCa, which was more apparent in the risk-based low-threshold pathway. Using a higher threshold for the risk-based pathway shows a greater biopsy avoidance and lower rates of iPCa detection, but with a small (3%) reduction in the overall detection of csPCa. All three pathways performed better than the “biopsy all” pathway in terms of avoiding unnecessary biopsies and overdiagnosis of iPCa. Decision curve analyses confirmed that at a threshold risk of 3.6-13.9% the risk-based low threshold pathway shows the overall highest net benefit.

The pooled negative predictive value of a negative MRI (PI-RADS score 1-2) is 90.8% for csPCa at a cut-off of ISUP grade group 2 and above,^3^ and can be further improved by the incorporation of PSA-density into biopsy decision making.^20^^,^^21^ However, NPV rates will depend on a number of factors, including image quality, radiologist experience, and cancer prevalence in the given patient population.^22^^,^^23^ Conversely, the positive predictive value and specificity of MRI show considerably more variation between centres, in part due to the threshold of radiologists to identify a positive lesion, and the variation in call rates of PI-RADS category 3 lesions.^24^^,^^25^ Models using PSA density to supplement MRI scores have shown promise, but consideration should also be given to adjusting biopsy thresholds depending on patient and/or clinician preference.^26^ This decision will be multifactorial and may be influenced by age, life expectancy, comorbidity, family history, and personal experience. Meta-analyses have shown the yield of csPCa in PI-RADS 3 targets to be 17%^27^; in our study, reducing this to 10.5% in the “low” risk group is of clear benefit and approaches the risk of missed csPCa in a negative (PI-RADS 1-2) MRI.^3^ Previous studies have shown that younger age (<65 years) and prior negative prostate biopsy alongside PSA density to be the strongest predictors of a low yield of csPCa in PI-RADS 3 targets^28^^,^^29^; however, this will also be affected by MRI quality and use of biparametric or multiparametric protocols,^22^^,^^30^ underlining the challenge. This supports the fact that a decision to avoid biopsy for lower-risk MRI findings (PI-RADS category 1-3) should be made on an individual patient basis and will depend on several factors including the priority to detect cancer or avoid the potentially harmful effects of biopsy.

Bittencourt et al. recently compared different diagnostic pathways including detection-based pathway (biopsy all), MRI focused pathway where MRI positive men undergo a combined targeted-systematic biopsy and a risk-based pathway in a cohort of biopsy-naïve men.^31^ They found that MRI-focussed and risk-based pathways showed the highest csPCa detection and a reduction in the diagnosis of iPCa, with the risk-based pathway having the highest potential to avoid biopsy.^31^ Our results are consistent with their recommendations and confirm that it is safe to avoid biopsies in low-risk patients. However, it is notable that our cohort showed a csPCa rate of 10.5% in low-risk patients (PSAD <0.10 ng mL^−2^) with PI-RADS category 3 change, just above the prevalence threshold for being within the Schoots et al. suggested category of “low risk”. This may be explained by the relatively low numbers of PI-RADS 3 in our cohort (10%), with the prevalence being more comparable to “4M study” rate of PI-RADS 3.^32^ Aside from this, all other categories of MRI and clinical risk matched with the proposed risk-based approach of Schoots et al. and serves to confirm that the risk-based model with different thresholds can give flexibility to users in applying risk-averse or biopsy averse strategies in decision making. The low PI-RADS 3 prevalence may reflect higher image quality at 3T and expert-level readers, and secondarily leading to a higher percentage yield of csPCa for this category.^23,^^33–35^ Notably this prevalence reduced to 8.8% (“low-risk”) in the PI-RADS v2.1 era, which may reflect a mixture of increased reader experience and/or improved image quality due to protocol changes or MRI software and hardware upgrades.^22,^^36–38^

PSA density >0.15 ng mL^−2^ is cited by both NICE and EAU guidelines as being indicative of higher risk.^39^^,^^40^ However, Pellegrino et al. have recently argued that this cutoff is justified only under the extreme scenario of poor MRI performance.^41^ Given improvements in MRI acquisition and interpretation, they suggest a higher PSA density cutoff of >0.20 ng mL^−2^ should be more generally applied and emphasize the need to consider this be varied based on the accuracy of prostate MRI and patient-specific factors such as digital rectal exam findings and prior biopsy results.^41^ Furthermore, Vickers et al. proposed that a threshold probability of 10% is reasonable, considering that a minimal number of urologists would prefer to conduct biopsies on over 10 patients to identify a single case of csPCa.^42^ Our study demonstrated that the risk-based low-threshold pathway has the highest net benefit at 10% threshold probability, suggesting that adapting a risk-based low-threshold pathway into the diagnostic pathway of PCa may be of clinical benefit.

Our study has some limitations, including the retrospective nature of the analysis. The cohort represents a single site, with patients scanned predominantly at 3 T and reported by experts, which may limit the generalizability of the results. Radiologists’ experience was not directly accounted for; however, all reporting radiologists were classified as “experts” in prostate MRI interpretation at the study entry point^13^^,^^33^^,^^43^ and accounting for dynamic changes in experience over the six-year study period would prove challenging. Further work should focus on including multiple centres and vendors and with radiologists of differing experience to confirm the consistency of results.

In conclusion, our study confirms the benefit of using risk stratification along with MRI in the prostate cancer diagnostic pathway to enable safe biopsy avoidance and limit the over-diagnosis of iPCa within a personalized medicine paradigm.

## Supplementary Material

tqad027_Supplementary_Data
