# Supplementary material for: Risk stratification of prostate cancer with MRI and prostate-specific antigen density-based tool for personalized decision making
Source: Br J Radiol. 2023 Dec 12;97(1153):113–9. doi: 10.1093/bjr/tqad027 (PMC11027333; doi:10.1093/bjr/tqad027)
Supplement: tqad027_Supplementary_Data [file tqad027_supplementary_data.docx]

**Supplemental Table 1:** Analysis of risk-based pathway in our population whose MRI were conducted until March 31, 2019:

|  | csPCa prevalence relative to  PI-RADS score | csPCa prevalence in the PSA- density risk groups | | | |
| --- | --- | --- | --- | --- | --- |
| **Total** | **ISUP ≥2 Ca prevalence** | **Low**  **(<0.10)** | **Intermediate – low**  **(0.10 - 0.15)** | **Intermediate- high**  **(0.15- 0.20)** | **High**  **(≥ 0.20)** |
| **ALL PI-RADS** | 317/1089  (29.1%) | 32/416  (7.7%) | 69/318  (21.7%) | 61/150  (40.7%) | 155/205  (75.6%) |
| **PI-RADS 1-2** | 26/619  (4.2%) | 6/323  (1.9%) | 6/197  (3.0%) | 7/63  (11.1%) | 7/36  (19.4%) |
| **PI-RADS 3** | 22/124 (17.7%) | 5/42  (11.9%) | 5/42  (11.9%) | 8/26  (30.8%) | 4/14  (28.6%) |
| **PI-RADS 4-5** | 269/346  (77.7%) | **21/51**  **(41.2%)** | **58/79**  **(73.4%)** | **46/61**  **(75.4%)** | **144/155**  **(92.9%)** |

| Very low | 0-5% csPCa (below population risk) | No biopsy |
| --- | --- | --- |
| Low | 5-10% csPCa (acceptable risk) | No biopsy |
| Intermediate-low | 10-20% csPCa | Consider biopsy |
| Intermediate-high | 20-30% csPCa | Highly consider biopsy |
| High | 30-40% csPCa | Perform biopsy |
| **Very high** | **>40% csPca** | **Perform biopsy** |

**Supplemental Table 2:** Analysis of risk-based pathway in our population whose MRI were conducted starting from April 1, 2019:

|  | csPCa prevalence relative to  PI-RADS score | csPCa prevalence in the PSA- density risk groups | | | |
| --- | --- | --- | --- | --- | --- |
| **Total** | **ISUP ≥2 Ca prevalence** | **Low**  **(<0.10)** | **Intermediate – low**  **(0.10 - 0.15)** | **Intermediate- high**  **(0.15- 0.20)** | **High**  **(≥ 0.20)** |
| **ALL PI-RADS** | 306/966  (31.7%) | 33/374  (8.8%) | 64/258  (24.8%) | 57/119  (47.9%) | 152/215  (70.7%) |
| **PI-RADS 1-2** | 8/494  (1.6%) | 1/279  (0.4%) | 3/144 (2.1%) | 2/37 (5.4%) | 2/34 (5.9%) |
| **PI-RADS 3** | 14/82 (17.1%) | 3/34 (8.8%) | 4/21 (19.0%) | 2/14 (14.3%) | 5/13 (38.5%) |
| **PI-RADS 4-5** | 284/390 (72.8%) | **29/61**  **(47.5%)** | **57/93 (61.3%)** | **53/68 (77.9%)** | **145/168 (86.3%)** |

| Very low | 0-5% csPCa (below population risk) | No biopsy |
| --- | --- | --- |
| Low | 5-10% csPCa (acceptable risk) | No biopsy |
| Intermediate-low | 10-20% csPCa | Consider biopsy |
| Intermediate-high | 20-30% csPCa | Highly consider biopsy |
| High | 30-40% csPCa | Perform biopsy |
| **Very high** | **>40% csPca** | **Perform biopsy** |
